# Sinus Node Dysfunction, Atrial Arrhythmias, and the Sinus Node Microcirculation

**DOI:** 10.1177/11795476221091409

**Published:** 2022-04-08

**Authors:** Susan K Fellner

**Affiliations:** Cell Biology and Physiology, University of North Carolina, Chapel Hill, NC 27599, USA

**Keywords:** microcirculation, sinus node dysfunction, atrial arrhythmia

## Abstract

A patient with sinus node dysfunction (SND) developed atrial arrhythmias that were abolished after avoidance of nonsteroidal anti-inflammatory medication and by the institution of a deliberate, modest increase in blood pressure, suggesting that there was a vascular component to the rhythm disturbance. Published anatomical evidence for a unique sinus node microcirculation substantiates the premise that SND may be sensitive to arterial flow. Because physicians have been unaware of the presence of a unique sinus node microcirculation, recognition of its presence may alter the approach to treatment of patients with SND and atrial arrhythmias, particularly in the elderly and those with diabetes.

## Introduction

Sinus node dysfunction (SND) or formerly sick sinus syndrome increases with aging, more than doubling for individuals aged 75 to 84 years compared to those a decade younger.^1^ Sinus node dysfunction (SND) is likely multifactorial in origin. Two recent reviews of SND and atrial arrhythmias emphasize the role of fibrosis in the structural and functional integrity of the sinus node (SN).^2,3^ Not considered in these reviews is the potential contribution of microvascular disease to the pathogenesis of SND.

## Case Report

The following case report is presented to illustrate the potential reversibility of atrial arrhythmias associated with SND and to raise awareness of the SN microcirculation.

An 84-year-old retired physician (nephrology) had a 7-year history of SND with relative bradycardia, premature atrial contractions (PACs), sinus pauses, and short runs of atrial tachycardia that interfered with exercise tolerance. Initial assessment included a stress test and echocardiogram, which were unremarkable. There was no evidence for coronary artery disease nor was there known chronic inflammation. Mild hypertension was controlled with losartan and hydrochlorothiazide. The patient began using nonsteroidal anti-inflammatory medications (naproxen 220 mg) daily for treatment of knee arthritis and she observed an increase in the frequency of arrhythmia thereafter. Cessation of the drug improved the rhythm disturbance and rechallenge caused resumption of the rhythm disturbance. The negative side effects of non-steroidal anti-inflammatory drugs (NSAIDs) in patients with cardiovascular disease are well known to clinicians. Inhibition of cyclo-oxygenase enzymes results in diminished production of prostacyclin, renal afferent arteriolar constriction, and subsequent salt retention. Reduction in prostacyclin causes lowered nitric oxide and endothelial cell dysfunction.^4^ Although these consequences of therapy with NSAIDs are recognized in patients with congestive heart failure, hypertension, and coronary artery disease, they have not previously been known to be associated with worsening SND and atrial arrhythmias.

The patient also noted that vasodilation from being in hot weather or while bathing was associated with a documented fall in blood pressure (100-105/60-65 mmHg) and the subsequent instigation of PACs, pauses, and runs of atrial tachycardia. An inadvertent increase in the dose of losartan likewise caused lower blood pressure (~110/65 sitting) and a marked increase in the frequency of the rhythm disturbance. Ambulatory ECG recordings during a 2-week period showed thousands of events. Heart rate varied from 44 to 180 beats per minute. The patient then adjusted her blood pressure medications and salt intake to keep the systolic blood pressure at about 130 to 135/75 mmHg. Thereafter she rarely experienced PACs. Resting heart rate rose from about 58 to approximately 68 beats per minute. Both exercise ability and heat tolerance improved considerably and have been sustained. Previous suggestions that she might require catheter ablation of an arrhythmogenic site or need a pacemaker were abandoned.

## Discussion

**Figure 1.**
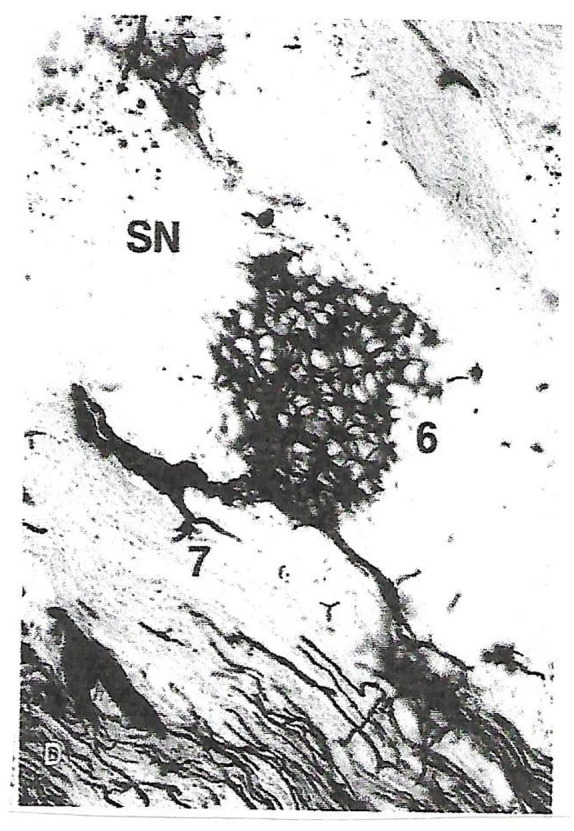
Human adult heart injected with India ink. SN is sinus node, 6 a glomerulus and 7, an afferent arteriole entering the glomerular capillary loop and a smaller diameter efferent arteriole exiting the structure (reprinted with permission, Petrescu et al^5^).

The patient’s positive clinical response to avoidance of NSAIDs and to a higher mean arterial pressure suggested to her that the sinus node (SN) might have a unique microcirculation that is particularly sensitive to changes in arterial perfusion. Her familiarity with the unique renal microcirculation stimulated this consideration. Despite the SN’s primacy as the pacemaker of the heart, surprisingly little is known about the microcirculation of the structure and how it differs from the adjacent atrium. Although it is well known that the sinus node artery arises from both coronary arteries, the arteriolar and capillary supply to the SN has been investigated in only a few studies. India ink injections of post-mortem normal human hearts demonstrated peripheral vascular networks of arterioles and capillary glomeruli that were different from the adjacent atrial myocardium.^5^ A large glomerular capillary loop with an afferent and efferent arteriole is clearly visible in the photomicrograph (Figure 1). This is an entirely novel finding. Glomeruli are well known in the kidney but have not been identified elsewhere in the human body. Post-mortem injections of human hearts of differing ages and sex showed a greater density of capillaries around the SN than in the adjacent atrial tissue.^6^ These studies give credence to the hypothesis that the SN has a unique structural and regulated microcirculation. Moreover, the afferent arteriolar vessels in the atrium may be particularly sensitive to changes in blood flow and to the physiologic effects of NSAIDs as is the case in the renal microcirculation.

The association of SND with atrial tachycardia, atrial ectopy, and atrial fibrillation is well recognized. The chronotropic incompetence of SND may permit firing of alternative impulses arising from the pulmonary vein or coronary sinus.^3^ In the case of this patient, improvement in resting heart rate as well as in the response to adrenergic stimulation with exercise likely diminished the emergence of ectopic beats.

The case study reported herein illustrates several important points. Relative hypotension, particularly in the setting of vasodilatation, was associated with chaotic atrial rhythm disturbances. Use of NSAIDs exaggerated the susceptibility to arrhythmia. Adrenergic stimulation during exercise and from a reduction in blood pressure itself did not result in an appropriate increase in sinus node driven impulses but did trigger output from other sites.

These clinical findings as well as previous anatomical evidence for a SN microcirculation with capillary glomeruli, support a potential critical role for the microcirculation of the SN. That the presence of a unique SN microcirculation has not been recognized by physicians previously supports a need to consider its implications and the potential impact of circulatory factors in caring for individuals with SND. Patients with diabetes and known microvascular disease may be particularly sensitive to reduced flow. Elderly patients, and especially those taking antihypertensive medications may likewise be at higher risk for SN hypoperfusion. Thus, the recognition of potentially reversible circulatory factor that may influence SN function may obvert interventional considerations.

## References

[1] Jensen PN Gronroos NN Chen LY , et al. Incidence of and risk factors for sick sinus syndrome in the general population. J Am Coll Cardiol. 2014;64:531-538. 25104519 10.1016/j.jacc.2014.03.056 PMC4139053

[2] Csepe TA Zhao J Hansen BJ , et al. Human sinoatrial node structure: 3D microanatomy of sinoatrial conduction pathways. Prog Biophys Mol Biol. 2016;120:164-178. 26743207 10.1016/j.pbiomolbio.2015.12.011 PMC4808362

[3] John RM Kumar S. Sinus node and atrial arrhythmias. Circulation. 2016;133:1892-1900. 27166347 10.1161/CIRCULATIONAHA.116.018011

[4] Patrono C. Cardiovascular effects of cyclooxygenase-2 inhibitors: a mechanistic and clinical perspective. Br J Clin Pharmacol. 2016;82:957-964. 27317138 10.1111/bcp.13048 PMC5137820

[5] Petrescu CI Niculescu V Ionescu N Vlad M Rusu MC. Considerations on the sinus node microangioarchitecture. Rom J Morphol Embryol. 2006;47:59-61. 16838059

[6] Ovcina F Cemerlić D. Clinical importance of intramural blood vessels in the sino-atrial segment of the conducting system of the heart. Surg Radiol Anat. 1997;19:359-363. 9479709 10.1007/s00276-997-0359-1

